# Highly pathogenic avian influenza management policy in domestic poultry: from reacting to preventing

**DOI:** 10.2807/1560-7917.ES.2024.29.42.2400266

**Published:** 2024-10-17

**Authors:** Timothée Vergne, Mathilde C Paul, Claire Guinat, Mattias Delpont, Brandon H Hayes, Sébastien Lambert, Jean-Pierre Vaillancourt, Jean-Luc Guérin

**Affiliations:** 1 IHAP, Université de Toulouse, INRAE, ENVT, Toulouse, France; 2 Faculty of Veterinary Medicine, University of Montreal, Saint-Hyacinthe, QC, Canada

**Keywords:** avian influenza, prevention, vaccination, density

## Abstract

The emergence of clade 2.3.4.4b H5N1 highly pathogenic avian influenza (HPAI) viruses in 2021 has led to unprecedented epidemics in poultry, changing epidemiological patterns of year-round infections in resident wild avifauna and more frequent spill-over events to mammals. Given this situation, it is important that we recognise that traditional HPAI management strategies are no longer sufficient, and policy changes are required. Poultry vaccination has emerged as a crucial intervention in the current control of HPAI, as evidenced by France's nationwide campaign targeting domestic ducks. However, due to the logistical challenges and potential trade implications of vaccination, broader structural reforms appear also necessary. These include a shift from farm-level to territorial-level biosecurity approaches, putting into practice the concept of ‘regional biosecurity’. Given the role duck farm density has played in successive HPAI epidemics in France, there is a need to think about the spatial distribution of poultry farms as a structural component of regional biosecurity and to consider the reduction of farm concentration as a measure to prevent viral spread. The integration of regional biosecurity and poultry vaccination into prevention strategies should impact the way poultry are produced and traded in the future.

## Background

Prior to 2021, highly pathogenic avian influenza (HPAI) viruses were introduced into Europe by migratory birds during the winter, leading to seasonal epidemic waves in domestic poultry. However, since the emergence of clade 2.3.4.4b H5N1 HPAI viruses in 2021, a remarkable shift in the epidemiology of the virus has occurred [1]. Infections are now reported throughout the year in the resident wild avifauna [2], leading to year-round spillover events and notable epidemics in domestic poultry. This change in epidemiological patterns has multiple ramifications. First, it raises important questions regarding wildlife conservation, particularly for vulnerable bird species. Between June and September 2023 alone [3], almost 500 H5N1 HPAI cases were reported in wild birds across Europe, with France, Germany, the Netherlands and the United Kingdom the hardest hit. In addition, there have been increasing reports worldwide of spill-over events to mammals such as foxes, ferrets, otters, bears, sea lions and dairy cattle, sometimes with unprecedented mass-mortality events [4]. Such repeated spill-overs increase the risk of viral adaptation to mammals, including humans [5]. Last but not least, clade 2.3.4.4b H5N1 HPAI viruses have had dramatic consequences for the poultry industry, with over 4,000 reported outbreaks in commercial poultry farms across 30 European countries during the epidemiological years 2021/22 and 2022/23 [3,6].

## Challenges to traditional management policies

Current HPAI management policies in disease-free countries are based on lessons learned from past epidemics. They emphasise early detection of outbreaks in poultry farms through careful clinical surveillance and contact tracing, reactive culling of infected flocks, quarantine or preventive culling of at-risk flocks and movement restrictions. While these strategies have successfully helped to control local HPAI epidemics for nearly two decades, they appear incapable of meeting the challenges posed by clade 2.3.4.4b HPAI viruses. The culling of millions of birds following massive outbreaks is a stark illustration of how these traditional strategies have failed to efficiently control the four HPAI (H5N8, H5N1) epidemics that have swept across Europe since the winter of 2016. Consequently, there is an urgent need for a paradigm shift in HPAI management policies in the poultry sector. Based on the recent experience in France, one of the most affected European countries, we are convinced that the key to improving the robustness of poultry production systems is through a combination of poultry vaccination and regional biosecurity.

## Vaccinating poultry

To date, mass vaccination against HPAI has mostly been used in some low- and middle-income countries, where implementing high on-farm biosecurity standards, early detection and timely depopulation of infected flocks pose considerable challenges [7]. In contrast, other countries, including in the European Union (EU), have not included vaccination in their toolkits to control HPAI due to concerns about trade restrictions and the potential for the virus to circulate silently within vaccinating countries [8]. However, it has become clear that in the future, it will be extremely challenging to control the risk of HPAI in Europe without vaccinating poultry [9]. The European Commission lifted restrictions on poultry vaccination against HPAI in February 2023 [10]. This policy shift is expected to drastically improve the ability of EU countries to control HPAI epidemics and reduce their impact on both poultry farmers and veterinary services.

To date, France is the only EU country to implement a nation-wide vaccination strategy, which started on 1 October 2023 [11]. This campaign, co-designed by the national veterinary services and private poultry sector stakeholders, focuses primarily on ducks, a species that has played an important role in recent epidemics [12]. Until May 2024, domestic ducks (excluding breeding ducks, which are vaccinated on a voluntary basis) had to be vaccinated with the subunit vaccine Volvac B.E.S.T. AI + ND (Boehringer Ingelheim, Ingelheim am Rhein, Germany), with two subcutaneous injections at 10 and 28 days of age, and a third dose at 56 days of age for ducks raised in high-risk zones during high-risk winter periods. In May 2024, the RESPONS AI H5 vaccine (Ceva Animal Health, Libourne, France), based on mRNA technology, also became available with two intra-muscular injections at 1 and 28 days of age. For both vaccines, a vaccinated flock is considered protected 1 week after the second injection, i.e. at 35 days of age. This programme is on-going with no end date defined at the time of publishing. The programme aims to reduce the susceptibility of ducks to HPAI and the level of virus shedding in infected vaccinated ducks. Reducing the infectious pressure exerted on other poultry flocks and the environment is expected to limit virus circulation in wild birds and mammals, and ultimately reduce the risk of emergence of zoonotic strains. To ensure efficient virus detection in vaccinated flocks, official surveillance plans need to be extremely robust and promptly integrate state-of-the-art surveillance protocols, including environmental sampling and rapid on-farm testing and sequencing. As of 10 months since the start of the vaccination campaign and up to July 2024, only 11 outbreaks have been reported across most types of poultry flocks in France (including one vaccinated duck flock, one incompletely vaccinated duck flock, one non-vaccinated breeder duck flock, six turkey flocks, one layer flock and one small multi-species poultry flock) (data not shown). This is in contrast to the 468 outbreaks reported in winter 2020/21 [13], 1,375 outbreaks in winter 2021/22 [13] and 382 outbreaks in winter 2022/23 [3]. Even though this reduction can also be due to other factors - such as a reduced circulation of HPAI viruses in Europe overall - a recent modelling study suggests that domestic duck vaccination has likely averted more than 250 outbreaks in poultry in France during winter 2023/24 [14].

However, given the logistical challenges incurred by the catching and vaccination of individual ducks multiple times, the cost of vaccination campaigns and their associated surveillance strategies (estimated at around 100 million euros for the first year [15]), combined with the international trade barriers that may arise due to poultry vaccination, relying on vaccination alone may not be a sustainable long-term solution. We therefore call for profound changes in the way the poultry industry is structured, to increase the robustness of poultry production systems against HPAI risk.

## Zooming out: from farm to regional biosecurity

Reducing HPAI risk is usually managed at the farm level by implementing rigorous on-farm biosecurity measures, including separating zones within farms, cleaning and disinfection protocols and regulating flows of animals, people and material [16]. However, numerous outbreaks have been reported on high-biosecurity farms, including breeding farms which have to comply with strict biosecurity protocols [6]. These outbreaks have highlighted the limits of traditional farm-level biosecurity approaches, prompting a re-evaluation of overall biosecurity strategies. We argue that biosecurity strategies need to incorporate a collective dimension by implementing a regional biosecurity approach. This would extend biosecurity principles beyond the boundaries of individual farms to tackle groups of farms within a territory, with the aim of reducing their effective contact rate (i.e. the rate of contact effectively leading to a transmission event). Whereas farm-level biosecurity is intended to manage infectious disease risk for individual farms, regional biosecurity aims at reducing risk at the collective level. As such, regional biosecurity standards can be fully integrated into regionalisation (or zoning) principles, by spatially-defining subpopulations with reduced disease risk in order to facilitate disease control or international trade. Regional biosecurity plans should include actions to promote the collective uptake of farm-level biosecurity standards, separate clean and dirty poultry traffic and implement go-forward principles, which involve starting with the least at-risk farms within a specific geographical area and ending with the most at-risk farms [17].

Due to the considerable impact that poultry farm density has on HPAI risk [18], we propose considering the spatial distribution of poultry farms as an essential component of regional biosecurity, with a priority focus on the riskiest species. A modelling study showed that reducing the density of duck farms in the top 20% of French municipalities in terms of duck farm density could have reduced the number of HPAI outbreaks threefold, increasing the robustness of the poultry sector to HPAI emergence [19]. In the short term, farm density can be reduced by limiting poultry placements during high-risk periods or by increasing the downtime between successive production cycles, as implemented in France during the 2022/23 epidemiological year under the initiative of the poultry industry [20]. In the long term, incentives could be introduced to promote the establishment of new production sites in territories with lower poultry farm density, or regulatory frameworks could define minimal distances between new and existing poultry farms. A less concentrated, but potentially wider, spatial distribution of poultry farms is expected to reduce the risk of an unmanageable surge of outbreaks in high-density zones, preventing veterinary services from becoming overwhelmed even if a wider region becomes affected. More research will be crucial to assess the epidemiological, sociological and economic synergies and trade-offs generated by these profound structural changes in diverse farming systems.

## Conclusion

Clade 2.3.4.4b HPAI viruses have clearly become adept at quickly overwhelming veterinary services and evading traditional reactive management strategies. To make the global poultry sector more robust, upgrading prevention strategies to include poultry vaccination and regional biosecurity measures should be a priority.
